# Sex differences in pain-related biopsychosocial assessments in patients with axial spondyloarthritis

**DOI:** 10.1186/s13075-025-03725-2

**Published:** 2026-01-09

**Authors:** Yvonne Maria van der Kraan, Davy Paap, Hans Timmerman, Freke Wink, Suzanne Arends, Michiel Reneman, Anneke Spoorenberg

**Affiliations:** 1 Department of Rheumatology and Clinical Immunology, University Medical Center Groningen, Groningen, the Netherlands; 2 Department of physiotherapy, Saxion University of Applied Sciences, Enschede, the Netherlands; 3 University Medical Center Groningen, Department of Anesthesiology, Pain Center, University of Groningen, Groningen, the Netherlands; 4 Frisius MC Leeuwarden, Rheumatology, Leeuwarden, the Netherlands; 5 University Medical Center Groningen, University of Groningen, Rehabilitation Medicine, Groningen, the Netherlands

**Keywords:** Axial spondyloarthritis, Pain, Biopsychosocial assessments, Central sensitization, Sex differences, Patient-reported outcomes

## Abstract

**Background:**

In axial spondyloarthritis (axSpA), (back)pain is the key symptom and often linked to inflammation. However, knowledge on sex-related differences in pain is limited. Therefore, our study aim was to explore sex differences in a broad spectrum of pain-related biopsychosocial assessments in patients with axSpA.

**Methods:**

Explorative cross-sectional study from Groningen Leeuwarden axSpA (GLAS) cohort. Following assessments were performed: pain-related questions 2–4 of Bath Ankylosing Spondylitis Disease Activity Index (BASDAI), quantitative sensory testing, including pain pressure threshold (PTT), temporal summation and conditioned pain modulation, and questionnaires assessing central sensitization, illness perception, coping strategies, anxiety/depression, and pain-related worrying. Effect sizes (ES) were interpreted as small (< 0.5), moderate (0.5–<0.8), or large (≥ 0.8). Univariable linear regression explored variables associated with BASDAI backpain question 2.

**Results:**

128 men and 73 women were included. Women scored higher on the BASDAI questions, had lower PPTs, higher CSI values (41.5 ± 13.6 vs. 30.6 ± 13.8; ES = 0.79) and used more often comforting cognitions (29.9 ± 4.1 vs. 27.8 ± 4.9; ES = 0.51), decreasing activity (21.0 ± 4.3 vs. 18.8 ± 4.5; ES = 0.49), and diverting attention (22.3 ± 3.9 vs. 20.1 ± 4.7; ES = 0.78) as coping strategies than men. Regression analysis demonstrated that factors associated with pain-related disease activity differ between sexes with varying R^2^.

**Conclusions:**

Our study suggests that men and women with axSpA show distinct patterns in pain-related biopsychosocial assessments. Women reported higher pain severity, different pain sensitivity, psychosocial profiles and associations with pain. These findings support the importance of addressing pain differently between sexes in clinical practice.

## Background

Chronic (back)pain is the main symptom of axial spondyloarthritis (axSpA) [1]. Despite treatment with anti-inflammatory therapy, such as biological disease modifying anti rheumatic drugs (bDMARDs), approximately 40% of axSpA patients continue to experience persistent pain, affecting health-related quality of life [2]. This presents a clinical challenge, as pain in axSpA patients may not be solely attributed to inflammation but may also involve non-nociceptive mechanisms and is bidirectionally related to various biopsychosocial factors, including pain thresholds and tolerance, illness perceptions, coping, depression and anxiety [3].

The International Classification of Diseases 11th Revision (ICD-11), has defined chronic primary pain (pain persisting or recurring for longer than three months) as a disease on its own, and not solely a symptom of another disease or disorder [4]. Central sensitisation (CS) is a non-nociceptive mechanism characterized by an increased responsiveness of nociceptive neurons in the central nervous system to normal input, leading to hyperalgesia (increased pain to painful stimuli), allodynia (pain from non-painful stimuli), and widespread pain [5, 6]. Currently, there is no gold standard for assessing CS, as it cannot be directly observed in humans due to the invasive nature of electrophysiological recordings required to measure central nociceptive neuron activity [6]. Quantitative sensory testing (QST) emerges as the most employed indirect method to objectively evaluate altered pain processing associated with CS in humans [6, 7]. QST encompasses pain pressure thresholds (PPT), temporal summation (TS) and conditioned pain modulation (CPM). Lower PPT and TS indicate pain hypersensitivity, while CPM reflects the effect of descending pain pathways, evaluating both facilitation and inhibition [7]. Furthermore, our recent study in axSpA showed that the Central Sensitization Inventory (CSI) can be used as an initial assessment questionnaire in clinical practice for identifying possible presence of CS [8].

In addition to the complex nature of chronic pain in axSpA, it is important to recognize that pain may manifest differently between men and women. This is also reflected in patient-reported outcomes (PRO), with women often reporting higher pain and disease activity scores than men [9–11]. There are no studies specifically exploring sex-related differences in QST in axSpA, but research in rheumatoid arthritis (RA) and osteoarthritis (OA) showed that women exhibit lower PPTs than men [12, 13]. Sex-related differences in TS and CPM are less consistent; some studies in healthy individuals suggest that women demonstrate higher TS and less CPM, whereas studies in RA and OA found no differences between sexes [12–16]. Two recent studies in axSpA found that sex was independently associated with CSI values, supporting a sex related effect on CS [8, 17]. Furthermore, widespread pain in axSpA is related to different outcomes across sexes: worse physical functioning, disease activity, anxiety and depression in men versus worse fear of movement and anxiety in women [18].

In addition to sensory processing, biological mechanisms may contribute to these differences. Testosterone has a protective effect on pain, while estrogen and progesterone increase variability in pain sensitivity through hormonal fluctuations [19]. Moreover, men have greater opioid receptor expression, driven by testosterone, enhancing analgesia [16]. In contrast, estrogen may reduce receptor availability and function, resulting in weaker and more variable pain relief in women [16].

Psychosocial factors such as pain coping strategies may also differ between men and women. In axSpA, women are more likely to use confrontation-based coping, whereas men tend to adopt avoidance strategies [20]. Additionally, women seem to be more prone to pain-related worrying than men [19, 21].

Sex differences in pain and disease burden in axSpA are increasingly recognized. Comprehensive biopsychosocial studies integrating both sensory and psychosocial pain assessments are lacking in axSpA, therefore our aim was to explore sex differences across a broad spectrum of pain-related biopsychosocial assessments in patients with axSpA.

## Methods

This study is an exploratory cross-sectional secondary analysis of data from a previously published study [8], with inclusion of axSpA outpatients from the Groningen Leeuwarden Axial Spondyloarthritis (GLAS) cohort. All patients had a clinical diagnosis of axSpA and fulfilled the ASAS classification criteria. Sex was extracted from the electronic record; gender identity was not assessed. No exclusion criteria were applied, as this study was based on a standard-of-care axSpA cohort. Follow-up visits were conducted in accordance with a standardized protocol. All methodological details are described in the original publication [8]. For this study, participants completed additional questionnaires: CSI, Revised Illness Perception Questionnaire (IPQ-R), Coping with Rheumatic Stressors questionnaire (CORS), Hospital Anxiety and Depression Scale (HADS) and Pain Catastrophizing Scale (PCS). On the same day as the outpatient visit and completing the questionnaires, QST was performed. Ethical approval for this study was obtained from the local ethics committees of the Frisius Medical Center and University Medical Center Groningen (UMCG) (RTPO 364/604). Written informed consent was obtained from each participant in accordance with the Declaration of Helsinki. The Strengthening the Reporting of Observational Studies in Epidemiology (STROBE) reporting guideline was followed.

### Pain-related assessments

Table 1 provides an overview of all assessments, including their scoring and interpretation.

**Table 1:** Overview of the pain-related assessments used in the study

Assessment	Description	Scoring/Interpretation
*PRO*		
BASDAI Q2	Axial pain (neck/back/hip) related to disease activity	NRS 0–10 0 = none, 10 = very severe
BASDAI Q3	Peripheral joint pain/swelling related to disease activity	NRS 0–10 0 = none, 10 = very severe
BASDAI Q4	Entheseal involvement related to disease activity	NRS 0–10 0 = none, 10 = very severe
CSI (part A)	Probability of central sensitisation	0–100 total score ≥ 40 = probable central sensitisation
IPQ-R	Illness perceptions (7 subscales)	0–30 per subscale Higher = heightened perception
CORS (Pain subscale)	Coping strategies (3 domains): comforting cognitions, decreasing activity, diverting attention	5–40 per domain Higher = heightened tendency for coping strategies
HADS	Anxiety and depression symptoms (2 subscales)	0–21 per subscale Higher = greater presence of symptoms related to anxiety or depression
PCS	Pain catastrophizing (pain-related worrying)	Score: 0–52 Higher = heightened tendency for pain-related worrying
*QST*		
PPT	Pressure at which sensation becomes painful	PPT sum: mean of 10 sites Lower score = more pain sensitivity
TS	Pain response after repeated stimuli	TS > 0 = pain facilitation
CPM	Inhibition of pain by conditioning stimulus (ice water test)	Negative = intact inhibition Positive = impaired inhibition

*PRO* Patient reported outcome, *BASDAI* Bath Ankylosing Spondylitis Disease Activity Index, *Q* Question, *CSI* Central Sensitization Inventory, *IPQ-R* Revised Illness Perception Questionnaire, *CORS* Coping with Rheumatic Stressors questionnaire, *HADS* Hospital Anxiety and Depression Scale, *PCS* Pain Catastrophizing Scale, *QST* Quantitative sensory testing, *PPT* Pain pressure threshold, *TS* temporal summation, *CPM* Conditioned pain modulation, *NRS* Numeric Rating Scale

QST was conducted by three assessors following a standardized protocol, including assessments of PPT, TS and CPM in a sequential design [22] based on the German Research Network on Neuropathic Pain (DFNS) protocol [22, 23] and the Nijmegen Aalborg Screening QST (NASQ) protocol [24–26].

PPT refers to the transition point of minimum pressure that induces an unpleasant sensation. PPT measurements were conducted using a handheld pressure algometer (Algometer Wagner FPX50, Greenwich, United States). Initially, a demonstration was performed on the dominant m. deltoid to familiarize the participant with the procedure. Subsequently, PPT was measured at ten sites: m. thenar bilateral, m. trapezius bilateral (at the level of the third thoracic vertebra, 4 cm laterally), m. rectus femoris bilateral (15 cm above the patella), m. abductor hallucis longus bilateral, reference area (opposite of the most painful area) and most painful area. Pressure was consistently applied at a rate of 5 N/s until the participant reported sensations of pain, burning, stinging, drilling, or pulling. The pressure level at which these sensations arose defined the participant’s PPT for that site. To obtain an overall mean for the PPT (PPT sum), PPT values from all sites were averaged [27]. Widespread and low PPT values serve as indicators of CS [5].

TS refers to the augmentation of increased pain perception when a sensory stimulus is administered repeatedly and swiftly over a brief duration, showing the presence of pain facilitation. TS was conducted using a PinPrick 256 mN, and an initial demonstration was carried out on the dominant forearm to familiarize the participant with the procedure. Subsequently, TS was measured at three sites: the non-dominant forearm, reference area and most painful area. TS procedure involved the delivery of a single stimulus, succeeded by a sequence of ten stimuli, and administered at a frequency of 1 second each, confined within the same skin area measuring 1 cm². Following initial stimulus and subsequently after the series of stimuli, the participant promptly assessed the perceived pain using a Visual Analogue Scale (VAS). This sequence was repeated a total of five times. The ‘wind-up’ phenomenon, indictive of CS, defined as TS values above zero, calculated as the mean of all five pain ratings for both the single stimuli and the successive 10 stimuli [28].

CPM refers to the phenomenon in which one pain-inducing stimulus can inhibit the perception of another pain, indicating impaired pain inhibition. CPM was conducted using an ice water bath test in a sequential design, which reflects descending inhibition pathways. CPM was evaluated at three sites: non-dominant m. rectus femoris, reference area and most painful area. Initially, a test stimulus was applied via a pressure algometer at a rate of 5 N/s until the participant reported a pain sensation corresponding to a pain score of 40 (on a scale of 0–100). Subsequently, a conditioning stimulus was administered by immersing the participant’s dominant hand into an ice water bath maintained at approximately + 5 °C for a maximum duration of 60 s. Immediately following the conditioning stimulus, the test stimulus was reapplied. The CPM outcomes were expressed as both percent change (relative CPM effect) and absolute values [29]. The relative CPM effect was calculated by subtracting initial pressure amount from the subsequent pressure amount, dividing by the baseline measurement, and then multiplying by 100%: 100*(CPM2-CPM1)/CPM2 [29]. Absolute differences were calculated by subtracting the initial pressure amount from the subsequent pressure amount. Negative values indicate intact endogenous pain inhibition, whereas positive values signify altered endogenous pain inhibition [29].

PRO included three disease activity questions from the Bath Ankylosing Spondylitis Disease Activity Index (BASDAI Q2–Q4), reflecting axial pain, peripheral joint pain/swelling, and entheseal involvement, respectively. BASDAI Q2 and Q3 are also included in the Axial Spondyloarthritis Disease Activity Score (ASDAS). Additional questionnaires were used to evaluate central sensitization (CSI) [30], illness perceptions (IPQ-R) [31], pain coping strategies (CORS) [32], anxiety and depression (HADS) [33], and pain catastrophizing (PCS) [34].

### Statistical analysis

Data analysis was performed using IBM SPSS Statistics for Windows Version 28.0.0 (IBM Corp., Armonk, NY). Descriptive statistics are presented as numbers of patients (%), mean (S.D.), and median (interquartile range; IQR p25-p75). Sex differences in patient and disease characteristics and pain-related biopsychosocial assessments were explored by group comparison of male and female participants using the Chi-Square test for categorical variables, Independent Samples T test for normally distributed continuous variables and Mann-Whitney U test for non-normally distributed continuous variables. Effect sizes (ES) were calculated using Cramér’s V for categorical variables, Cohen’s d for normally distributed variables, and rank-biserial correlation for non-normally distributed data [35]. ES were interpreted as small (< 0.5), moderate (0.5–<0.8), or large (≥ 0.8) [35]. 95% CIs were reported for mean and proportion differences. Violin plots were created to visualize QST measurements in male and female participants. Additionally, univariable linear regression analyses were performed for BASDAI Q2 and Q3 stratified by sex, with explained variances expressed as R^2^, if score variability was sufficient. Missing data were not imputed; for each variable analyses were performed based on available cases.

## Results

### Patient and disease characteristics

A total of 201 patients were included, 128 (63%) men and 73 (37%) women. Almost all patients were Caucasian. Most patient and disease characteristics were similar between sexes, except that men were more frequently classified with radiographic axSpA (r-axSpA) and had more often a positive HLA-B27 status than women (72% vs. 51% and 84% vs. 68%, respectively), while women had higher BMI than men (mean 29.0 ± 6.9 vs. 27.2 ± 4.9) (Table 2).

**Table 2:** Patient and disease characteristics stratified for sex (*n* = 201)

	Total group (*n* = 201)	Men (*n* = 128)	Women (*n* = 73)
Age, years	51.6 [38.5–58.8]	50.8 [38.2–59.2]	51.6 [39.3–58.4]
BMI, kg/m^2^	27.8 ± 5.8	27.2 ± 4.9	29.0 ± 6.9
Current smoker	51 (26%)	34 (27%)	17 (24%)
High education level^a^	68 (34%)	47 (37%)	21 (29%)
*Occupational status*			
Working Not working Studying Retired Not capable due to axSpA	126 (63%) 18 (9%) 5 (3%) 18 (9%) 32 (16%)	86 (68%) 5 (4%) 3 (2%) 12 (10%) 20 (16%)	40 (55%) 13 (18%) 2 (3%) 6 (8%) 12 (16%)
Symptom duration, years	13.0 [5.0–24.0]^2^	13.0 [5.0–25.0]^2^	12.5 [5.3–23.0]^2^
Diagnostic delay, years	5.0 [1.0–12.0]^2^	5.0 [1.0–12.0] ^2^	6.0 [2.0–15.0]^2^
*SpA features*			
Classified as r-axSpA	129 (64%)	92 (72%)	37 (51%)
HLA-B27 positive	153 (78%)	104 (84%)	49 (68%)
Current peripheral arthritis^b^	6 (3%)^1^	3 (2%)^1^	3 (5%)^1^
History of IBD	16 (8%)	12 (10%)	4 (6%)
History of uveitis	52 (26%)	34 (27%)	18 (25%)
History of psoriasis	13 (7%)	10 (8%)	3 (4%)
*Medication use*			
NSAID use	117 (59%)	76 (61%)	41 (57%)
bDMARD use	104 (53%)	64 (51%)	40 (56%)
*Disease activity assessments*			
ASDAS ASDAS ≥ 2.1	2.1 ± 0.9 98 (51%)	1.9 ± 0.9 52 (41%)	2.4 ± 1.0 46 (63%)
BASDAI, 0–10	3.9 ± 2.2	3.4 ± 2.1	4.7 ± 2.1
CRP, mg/l	2.0 [1.0–4.2.0.2]	1.8 [1.0–4.0]	2.5 [1.0–6.0]
*Patient reported outcomes*			
ASQOL, 0–18	5.4 ± 4.8	4.2 ± 4.6	7.5 ± 4.4
BASFI, 0–10	3.3 ± 2.4	3.0 ± 2.3	3.8 ± 2.4

Values are presented in: n (%), mean (S.D.) or median (IQR). All % values exclude missing items for their respective characteristic. All missing values < 5% unless otherwise specified: ^1^5–10% missing; ^2^11–15% missing

^a^Defined as International Standard Classification of Education (ISCED) level > 5. ^b^ Defined as a swollen joint count of ≥ 1

*BMI* Body mass index, *axSpA* axial spondyloarthritis, *SpA* spondyloarthritis, *r-axSpA* radiographic axial spondyloarthritis, *HLA-B27* Human leukocyte antigens B27, *IBD* Inflammatory Bowel Disease, *NSAID* Non-steroidal anti-inflammatory drug, *bDMARD* biologic disease modifying anti-rheumatic drug, *ASDAS* Ankylosing Spondylitis Disease Activity Score, *BASDAI* Bath Ankylosing Spondylitis Disease Activity Index, *CRP* C-reactive protein, *ASQOL* Ankylosing spondylitis quality of life, *BASFI* Bath ankylosing spondylitis functional index

Concerning disease activity and axSpA-related PROs, women had overall higher scores than men. For example, mean ASDAS was 2.4 ± 1.0 in women and 1.9 ± 0.9 in men. However, median CRP levels did not differ. Women also reported higher scores on disease-related QoL (ASQoL) and physical functioning (BASFI) than men, meaning lower disease-related QoL and physical functioning (Table 2).

### Pain related to disease activity assessments

Women reported higher scores than men on the axial and peripheral pain, and entheseal involvement questions related to disease activity (Table 3). For axial pain (BASDAI Q2), the mean score was 5.4 ± 2.5 in women vs. 4.0 ± 2.5 in men (ES = 0.55). For peripheral pain (BASDAI Q3), scores were 4.0 ± 2.9 vs. 2.6 ± 2.5 (ES = 0.49). For entheseal discomfort (BASDAI Q4), the mean score was 4.3 ± 2.9 in women vs. 2.5 ± 2.6 in men (ES = 0.64).

**Table 3:** Pain-related patient reported assessments stratified for sex (*n* = 201)

	Men (*n* = 128)	Women (*n* = 73)	*P* value	Mean difference (95% CI)	Effect size	
*Pain related to disease activity*						
BASDAI Q2^a^	4.0 ± 2.5	5.4 ± 2.5	< 0.001	1.4 (−2.15, −0.65)	0.55	
BASDAI Q3^b^	2.6 ± 2.5	4.0 ± 2.9	< 0.001	1.4 (−2.15, −0.65)	0.49	
BASDAI Q4^c^	2.5 ± 2.6	4.3 ± 2.9	< 0.001	1.8 (−2.55, −0.94)	0.64	
*Central sensitization*						
CSI total score (0–100) CSI ≥ 40	30.6 ± 13.8 35 (27%)	41.5 ± 13.6 46 (63%)	< 0.001 < 0.001	10.9 (−14.86, −6.89) 36% (−0.48, −0.22)	0.79 0.36	
*Illness perception*						
IPQ-R Identity, 0–14 Timeline acute/chronic, 6–30 Timeline cyclical, 4–20 Personal control, 6–30 Treatment control, 6–30 Illness coherence, 5–25 Consequences, 6–30 Emotional representations, 6–30	3.0 [2.0–5.0] 28.0 [26.0–29.0] 13.8 ± 3.8 20.8 ± 4.4 18.2 ± 3.3 18.8 ± 4.3 15.9 ± 5.2 12.0 [10.0–15.5.0.5]	4.0 [3.0–6.0] 28.0 [25.0–30.0] 14.7 ± 3.4 20.7 ± 3.8 18.2 ± 3.0 19.6 ± 4.1 17.4 ± 5.4 13.0 [11.0–15.0]	0.021 0.984 0.124 0.814 0.848 0.239 0.059 0.617	1.0 (−1.28, 0.05) 0.0 (−0.86, 1.27) 0.9 (−1.90, 0.23) −0.1 (−1.08, 1.37) 0.0 (−0.83, 1.01) 0.8 (−1.97, 0.50) 1.5 (−3.04, 0.07) 1.0 (−1.15, 0.88)	0.25 0.06 0.23 0.04 0.03 0.17 0.28 0.04	
*Coping with pain*						
CORS pain Comforting cognitions, 9–36 Decreasing activity, 8–31 Diverting attention, 8–30	27.8 ± 4.9 18.8 ± 4.5 20.1 ± 4.7	29.9 ± 4.1 21.0 ± 4.3 22.3 ± 3.9	0.002 < 0.001 0.001	2.1 (−3.45, −0.76) 2.2 (−3.55, −0.98) 2.2 (−3.46, −0.87)	0.51 0.49 0.78	
*Anxiety and depression*						
HADS Anxiety, 0–21 Depression 0–21	4.0 [2.0–6.0] 6.0 [3.0–9.0]	4.0 [2.0–7.0] 6.0 [3.0–8.0]	0.324 0.439	0.0 (−1.38, 0.51) 0.0 (−0.68, 1.52)	0.14 0.11	
*Pain-related worrying*						
PCS, 0–52	11.0 [5.0–20.0]	14.0 [5.0–19.5.0.5]	0.551	3.0 (−3.12, 2.42)	0.04	

Values are presented in: mean (S.D.) or median [IQR]. Categorical variables were tested using the Chi-square test; normally distributed continuous variables using the Independent Samples t-test; and non-normally distributed continuous variables using the Mann–Whitney U test

^a^’How would you describe the overall level of axSpA neck, back, or hip pain you have had?’. ^b^’How would you describe the overall level of pain/swelling in joints other than neck, back, hips you have had?’. ^c^’How would you describe the overall level of discomfort you have had from any areas tender to touch or pressure?’

*CI* Confidence Interval, *BASDAI* Bath Ankylosing Spondylitis Disease Activity Index, *CSI* Central Sensitization Inventory, *IPQ-R* Revised Illness Perception Questionnaire, *CORS* Coping with Rheumatic Stressors questionnaire, *HADS* Hospital Anxiety and Depression Scale, *PCS* Pain Catastrophizing Scale

### Sensory processing: QST measurements

**Fig. 1:**
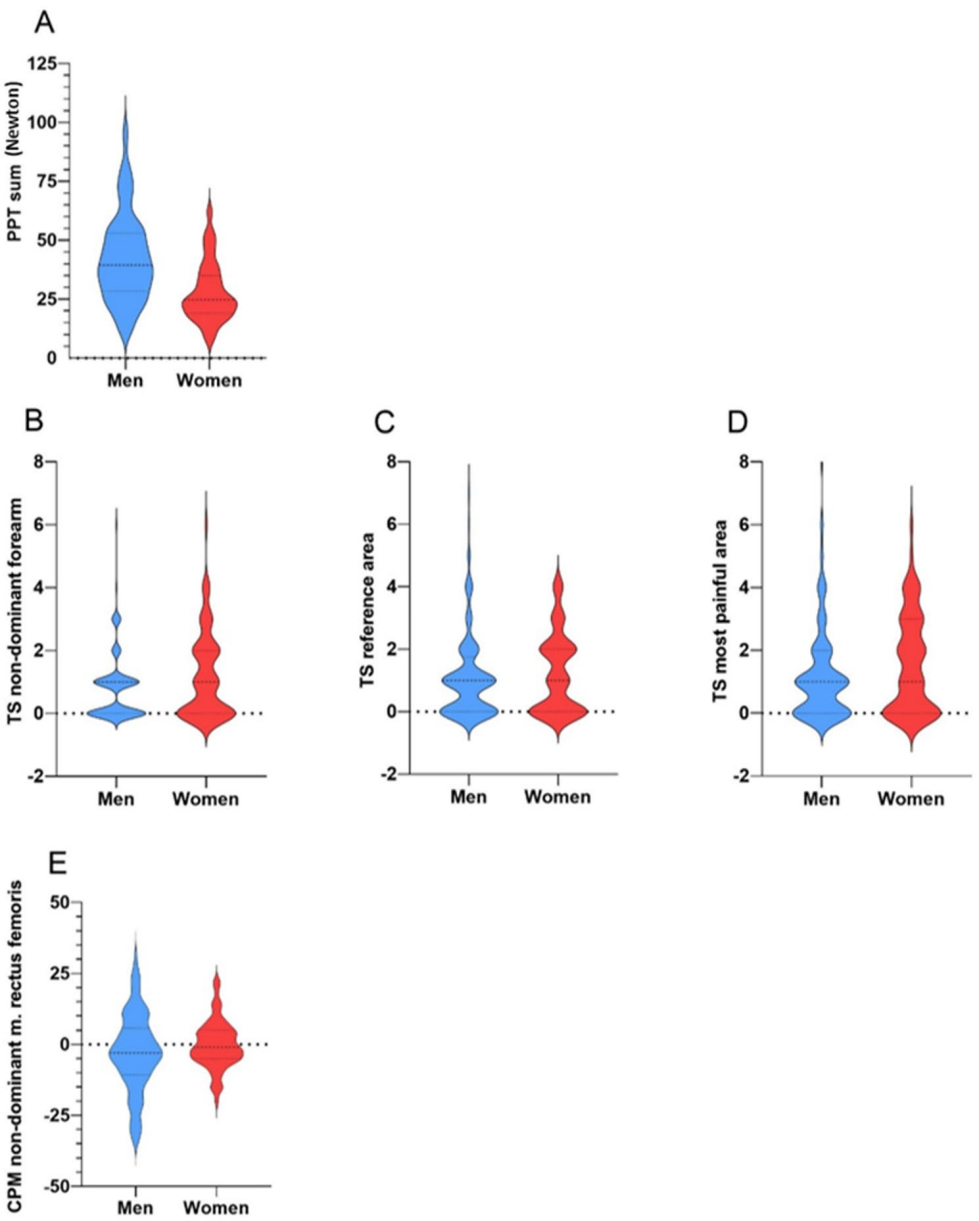
Quantitative sensory testing measurements stratified for sex (*n* = 201). **A**: Pain pressure threshold (PPT) sum stratified for sex; **B**: Temporal summation (TS) at the non-dominant forearm stratified for sex; **C**: Temporal summation (TS) at the reference area stratified for sex; **D**: Temporal summation (TS) at the most painful area stratified for sex; **E**: Conditioned pain modulation (CPM) at the non-dominant musculus rectus femoris stratified for sex

Women demonstrated lower PPTs at all sites than men with moderate to large ES ranging from 0.54 to 1.01 (mean PPT at non-painful sites 29.0 ± 14.6 vs. 43.4 ± 21.3, mean PPT at reference area 25.0 ± 13.8 vs. 38.6 ± 22.1, median PPT at most painful area 19.8 [13.0–28.7.0.7] vs. 32.2 [22.4–51.6]), indicating higher pain sensitivity in women in relation to men. Furthermore, women showed slightly higher TS values than man with only small ES ranging from 0.14 to 0.32, suggesting somewhat enhanced pain facilitation compared to men (Table 4). CPM values, representing descending inhibition pathways, were similar between men and women with small ES of 0.22 (Table 4). See Fig. 1 for differences in PPT, TS and CPM stratified for sex.

**Table 4:** Sensory processing: QST measurements stratified for sex (*n* = 201)

		Men (*n* = 128)	Women (*n* = 73)	*P* value	Mean difference (95% CI)	Effect size
PPT thenar m. trapezius m. rectus femoris m. abductor hallucis reference area^a^ painful area	left right left right left right left right	37.4 ± 17.5 40.6 ± 19.1 40.1 ± 22.8 39.4 ± 20.9 58.0 ± 25.1 55.9 ± 26.8 37.0 ± 18.4 38.6 ± 19.8 38.6 ± 22.1 32.2 [22.4–51.6]	26.1 ± 12.1 29.0 ± 14.2 26.8 ± 13.5 26.8 ± 14.5 35.2 ± 17.9 32.1 ± 16.3 27.5 ± 13.2 28.7 ± 15.1 25.0 ± 13.8 19.8 [13.0–28.7.0.7]	< 0.001 < 0.001 < 0.001 < 0.001 < 0.001 < 0.001 < 0.001 < 0.001 < 0.001 < 0.001	11.3 (7.12, 15.41) 11.6 (6.95, 16.67) 13.3 (8.31, 18.40) 12.6 (7.62, 17.53) 22.8 (16.75, 28.77) 23.8 (17.83, 29.82) 9.5 (5.04, 13.91) 9.9 (4.94, 14.80) 13.6 (8.66, 16.65) 12.4 (9.74, 19.68)	0.71 0.67 0.67 0.67 1.00 1.01 0.57 0.54 0.70 0.75
TS non-dominant forearm TS = 0 TS > 0 reference area^a^ TS = 0 TS > 0 painful area TS = 0 TS > 0		0.7 [0.1–1.4] 61 (48%) 67 (52%) 0.8 [0.2–1.9] 50 (39%) 78 (61%) 0.9 [0.2–2.1] 48 (38%) 80 (62%)	0.7 [0.2–2.1] 31 (42%) 42 (58%) 1.1 [0.2–2.2] 24 (33%) 49 (67%) 1.3 [0.3–2.7] 22 (30%) 51 (70%)	0.077 0.660 0.660 0.347 0.730 0.730 0.062 0.595 0.595	0.0 (−0.71, −0.02) 6% (−0.09, 0.19) −6% (−0.19, 0.09) 0.3 (−0.54, 0.20) 6% (−0.08, 0.19) −6% (−0.19, 0.08) −0.4 (−0.82, 0.04) 8% (−0.06, 0.20) −8% (−0.20, 0.06)	0.32 0.05 0.05 0.14 0.06 0.06 0.26 0.07 0.07
CPM non-dominant m. rectus femoris negative CPM positive CPM		2.8 ± 13.5 (−2%) 82 (64%) 46 (36%)	0.2 ± 8.3 (2%) 40 (55%) 33 (45%)	0.088 0.645 0.645	2.6 (−0.40, 5.71) 9% (−0.05, 0.23) −9% (−0.23, 0.05)	0.22 0.09 0.09

Values are presented in: mean ± S.D. or median [IQR]. Categorical variables were tested using the Chi-square test; normally distributed continuous variables using the Independent Samples t-test; and non-normally distributed continuous variables using the Mann–Whitney U test. PPT is presented in Newton (N); TS and CPM are unitless derived change scores without a fixed scale or range. CPM is presented in absolute and relative (%) values

*QST* Quantitative Sensory Testing, *CI* Confidence Interval, *PPT* Pain Pressure Threshold, *TS* Temporal Summation, *CPM* Conditioned Pain Modulation

### Pain-related patient reported assessments

Women had higher CSI scores than men (mean 41.5 ± 13.6 vs. 30.6 ± 13.8; ES 0.79), with more women scoring above the general (63% vs. 27%; ES 0.76) and sex-specific cut-off values for the CSI (75% vs. 64%; ES 0.36). Differences in pain coping strategies showed also moderate ES, with women relying more on comforting cognitions (mean 29.9 ± 4.1 vs. 27.8 ± 4.9, ES = 0.51), decreasing activity (21.0 ± 4.3 vs. 18.8 ± 4.5, ES = 0.49), and diverting attention (22.3 ± 3.9 vs. 20.1 ± 4.7, ES = 0.78) than men. ES in illness perception (IPQ), anxiety and depression (HADS) and pain related worrying (PCS) were small (Table 3).

### Biopsychosocial variables associated to disease activity reported backpain

In univariable linear regression analyses, high educational level was significantly associated with lower BASDAI Q2 scores in men (R²=0.16, B=−2.08), but not in women (R²= 0.07, B = 0.36) (Table 5). In women, a r-axSpA diagnosis was more strongly associated with lower BASDAI Q2 scores (R²=0.08, B=–1.41) than in men (R²=0.15, B=–0.84). No statistical evidence of association was found for CRP levels with BASDAI Q2 scores in neither men (R²=0.09, B = 0.04) nor women (R²=0.03, B = 0.03). Furthermore, attributing symptoms to axSpA (IPQ-R identity) explained more variance in BASDAI Q2 scores in men (R²=0.32, B = 0.55) than in women (R²=0.17, B = 0.51). Perceived treatment efficacy (IPQ-R treatment control) was associated with lower BASDAI Q2 scores in men (R²=0.10, B=−0.24), but there was no statistical evidence of association in women (R²=0.00, B = 0.01). Likewise, beliefs about the impact of axSpA on daily life (IPQ-R consequences) were more relevant and somewhat stronger associated to higher BASDAI Q2 scores in men (R²=0.24, B = 0.24) than in women (R²=0.05, B = 0.11). Differences in associations with PPT were (very) small. Due to low scores and limited variability in BASDAI Q3, only BASDAI Q2 results are reported.

**Table 5:** Univariable linear regression stratified by sex for BASDAI Q2^a^ with patient, disease and pain-related assessments

	Male participants (*n* = 128)			Female participants (*n* = 73)		
	*R* ^2^	B (95% CI)	*p*	*R* ^2^	B (95% CI)	*p*
Age, years	0.00	0.01 (−0.03, 0.04)	0.718	0.01	−0.02 (−0.07, 0.03)	0.392
BMI, kg/m^2^	0.02	0.08 (−0.03, 0.27)	0.089	0.00	−0.00 (−0.09, 0.09)	0.926
High level of education	0.16	−2.08 (−2.94, −1.22)	< 0.001	0.07	0.36 (−0.98, 1.71)	0.591
Working/student	0.05	−1.20 (−1,18, −0.21)	0.017	0.06	−1.23 (−2.43, −0.03)	0.045
Smoking	0.01	0.63 (−0.38, 1.65)	0.219	0.00	0.18 (−1.24, 1.60)	0.797
Symptom duration	0.04	−0.01 (−0.05, 0.03)	0.701	0.07	−0.06 (−0.12, −0.00)	0.046
Clinical diagnosis r-axSpA	0.15	−0.84 (−1.85, 0.17)	0.104	0.08	−1.41 (−2.58, −0.02)	0.019
CRP, mg/l	0.09	0.04 (−0.03, 0.01)	0.287	0.03	0.03 (−0.09, 0.15)	0.641
*Medication use*						
NSAID use	0.01	0.49 (−0.43, 1.40)	0.292	0.03	0.93 (−0.29, 2.14)	0.131
bDMARD use	0.01	0.35 (−0.56, 1.25)	0.452	0.00	0.02 (−1.22, 1.26)	0.974
*QST measurements*						
PPT sum values, N	0.02	−0.02 (−0.04, 0.01)	0.113	0.08	−0.06 (−0.10, −0.01)	0.016
TS non-dominant forearm, N	0.00	0.05 (−0.39, 0.50)	0.820	0.00	0.04 (−0.45, 0.53)	0.866
CPM non-dominant m. rectus femoris, N	0.03	−0.03 (−0.07, −0.00)	0.047	0.02	0.04 (−0.03, 0.12)	0.245
*Psychosocial assessments*						
CSI, 0–100	0.27	0.09 (0.07, 0.12)	< 0.001	0.32	0.10 (0.07, 0.14)	< 0.001
IPQ-R						
identity, 0–14	0.32	0.55 (0.42, 0.69)	< 0.001	0.17	0.51 (0.24, 0.79)	< 0.001
timeline acute/chronic, 6–30	0.06	0.17 (0.05, 0.29)	0.008	0.00	−0.03 (−0.19, 0.12)	0.673
timeline cyclical, 4–20	0.10	0.21 (0.10, 0.33)	< 0.001	0.00	0.04 (−0.14, 0.22)	0.649
personal control, 6–30	0.06	−0.14 (−0.24, −0.03)	0.009	0.00	−0.03 (−0.20, 0.13)	0.684
treatment control, 6–30	0.10	−0.24 (−0.37, −0.11)	< 0.001	0.00	0.01 (−0.20, 0.22)	0.926
illness coherence, 5–25	0.03	−0.10 (−0.21, 0.00)	0.050	0.09	−0.19 (−0.34, −0.05)	0.011
consequences, 6–30	0.24	0.24 (0.16, 0.32)	< 0.001	0.05	0.11 (−0.00, 0.22)	0.057
emotional representations, 6–30	0.14	0.26 (0.14, 0.38)	< 0.001	0.06	0.29 (0.01, 0.36)	0.039
CORS pain						
comforting cognitions, 9–36	0.04	−0.10 (−0.19, −0.01)	0.038	0.02	−0.08 (−0.23, 0.07)	0.298
decreasing activities, 8–31	0.13	0.20 (0.10, 0.30)	< 0.001	0.00	0.03 (−0.11, 0.17)	0.703
diverting attention, 8–30	0.00	−0.00 (−0.10, 0.09)	0.929	0.00	−0.00 (−0.17, 0.16)	0.949
HADS anxiety, 0–21	0.06	0.18 (0.04, 0.32)	0.012	0.09	0.23 (0.06, 0.41)	0.011
HADS depression, 0–21	0.12	0.23 (0.12, 0.34)	< 0.001	0.12	0.24 (0.08, 0.40)	0.004
PCS, 0–52	0.20	0.11 (0.07, 0.15)	< 0.001	0.20	0.13 (0.07, 0.20)	< 0.001

^a^’How would you describe the overall level of axSpA neck, back, or hip pain you have had?’

*BASDAI* Bath Ankylosing Spondylitis Disease Activity Index, *r-axSpA* radiographic axial spondyloarthritis, *CRP* C-reactive protein, *NSAID* Non-steroidal anti-inflammatory drug, *bDMARD* biologic disease modifying anti-rheumatic drug, *QST* Quantitative sensory testing, *PPT* Pain pressure threshold, *TS* Temporal summation, *CPM* Conditioned pain modulation, *CSI* Central sensitisation inventory, *IPQ-R* Revised Illness Perception Questionnaire, *CORS* Coping with Rheumatic Stressors questionnaire, *HADS* Hospital Anxiety and Depression Scale, *PCS* Pain Catastrophizing Scale

## Discussion

In our cross-sectional standard-of-care axSpA cohort, observed sex differences in patient and disease characteristics are consistent with those reported in other cohort studies, including higher PRO scores in women, such as the patient-reported part of ASDAS and BASDAI [11, 12, 32–35].

Unique to the present study is the exploration of sex differences in broad spectrum of biopsychosocial assessments related to (chronic) pain including two disease activity-related pain questions incorporated in both the BASDAI and ASDAS. In our cohort, women reported higher spinal and peripheral pain, and entheseal discomfort than men. Because patients were treated according to the international guidelines and received anti-inflammatory treatment when indicated, CRP levels were low in almost all patients and peripheral arthritis was negligible in both sexes. This implies that axial (BASDAI Q2) and peripheral pain (BASDAI Q3), and entheseal discomfort (BASDAI Q4) in this cohort was not primarily driven by current active inflammation, and could instead be of nociplastic origin. This interpretation aligns with previous research demonstrating that women with axSpA report more widespread pain than men, which is associated with higher disease activity scores reported and poorer health-related outcomes [18]. Together, these findings suggest that factors beyond inflammation, such as CS and pain-related psychosocial factors, may influence BASDAI and ASDAS differently by sex.

In our earlier study with structural equation modelling (SEM), illness perception showed the strongest direct association with ASDAS, alongside CSI scores, BMI, and physical activity [36]. These factors may differ by sex, as supported by our current regression findings revealing different biopsychosocial associations with axial pain (BASDAI Q2) in men and women. Although BMI was previously linked to ASDAS in our SEM analyses, it was not associated with axial pain (BASDAI Q2) in the current study. While BMI may influence pain perception [37], the small difference in BMI between sexes in our study, is unlikely to explain the observed sex differences in pain outcomes.

To the best of our knowledge, this is the first study assessing sex-related differences in a broad spectrum of pain measures in axSpA, and overall a different pattern in men and women seems to emerge. Women had lower PPTs at all sites, indicating increased nociceptive sensitivity. This aligns with findings in RA and OA [12, 13]. These lower PPTs in women may indicate increased peripheral sensitization, particularly due lower thresholds at the most painful area and reference area compared to men. However, since this pattern appeared across all sites, it likely also reflects CS, which may coexist with peripheral sensitization. However, these lower PPTs in women do not necessarily mean that there are differences in central pain pathways between sexes. PPTs may also be influenced by psychosocial factors that modulate pain perception [38].

Our study found somewhat higher TS values in women. TS reflects nociceptive facilitation, where repeated stimuli increase pain perception. Studies in OA patients and healthy individuals [12, 39] found also higher TS in women, possibly due to greater C-fiber involvement, which are responsible for transmitting signals related to potential tissue damage, commonly perceived as slow, dull pain [40]. However, a study in RA found no significant sex differences in TS, highlighting inconsistencies across different conditions which needs further research [13].

No sex differences were observed in CPM, which assesses endogenous pain modulation via descending inhibitory pathways, which aligns with a study in RA [13, 41]. Prior research showed that sex and age explain only a small part of CPM variance [13, 41]. Therefore, the absence of sex differences in CPM in our study may reflect high inter-individual variability rather than similar pain modulation between sexes. Psychological and genetic factors in individuals probably play a larger role in this inter-individual CPM variability [42, 43].

The pain-related patient reported assessments showed that women had higher CSI values than men, and women associated with CS. These results align with findings in musculoskeletal and chronic pain disorders [44–46]. This difference was expected, as women generally report higher pain severity, lower pain thresholds, and less tolerance to noxious stimuli than men, as was also observed in the present study [8, 9, 17, 21]. Additionally, chronic pain conditions linked to CS, such as fibromyalgia, are more prevalent in women [47]. Given that our previous study showed that CSI captures both CS and related psychosocial factors [8], it can be hypothesized that higher CSI values in women may also reflect a greater psychosocial burden influencing their pain perception and experience.

Illness perceptions and disease-related cognitive and emotional representations influence well-being by shaping cognitions, emotions and body sensations, such as pain. In our study, illness perceptions were generally similar between men and women, with low overall scores indicating no clinically relevant differences. However, univariable linear regression analysis showed that attributing symptoms to axSpA, perceived treatment efficacy, and beliefs about the impact of axSpA were more strongly associated with pain-related disease activity in men than in women. This suggests that while illness perceptions may be similar, their relevance to pain-related disease activity can differ by sex. Results on sex differences in illness perceptions in rheumatic and musculoskeletal diseases (RMDs) are not conclusive. A study in RA found sex differences in most illness perception domains of the IPQ-R [48]. This discrepancy may be due to differences in patient populations, as axSpA typically affects younger individuals with a fluctuating disease course, while RA affects older and especially female individuals with a more chronic disease course, leading to for example varying perceptions of illness severity, treatment, and coping strategies. Additionally, validity of IPQ-R in RMDs still needs to be established [49].

Our results indicate that women used comforting cognitions, decreasing activity, and diverting attention as coping strategies more frequently than men. Coping strategies likely reflect potential impact of societal expectations, roles and gender norms [50]. Gender is a multifaceted and complex concept shaped by society, culture and psychology, and defines roles and behaviours [51]. In our study, societal expectations shaped by Western cultural norms may have influenced observed differences in coping strategies. Men are often socialized to handle pain with stoicism and self-reliance, while women are encouraged to seek support and express emotions more openly [16, 50].

Depression and anxiety are known to influence pain perception, with women generally experiencing higher levels of pain-related worrying, anxiety, and depression than men in the general population [52]. Additionally, widespread pain is associated with worse HADS anxiety and depression in axSpA [18]. In our study, no sex differences in HADS scores were found, possibly due to relatively long disease duration leading to a certain amount of resignation and acceptation, effective previous treatments, and overall low HADS scores limiting detection of differences.

A strength of our study is the integration of QST, CSI, and pain-related patient reported assessments in a well-defined cohort, offering insights into sex-related differences and biopsychosocial factors in pain within axSpA. Notably, this is also the first study to explore sex-related differences in CS within an axSpA population, with a large, diverse sample of treated patients, making our findings relevant to future research and clinical practice. Our findings support the hypothesis that pain is associated with individual biopsychosocial factors and may require a personalized approach. Men and women showed differences in the factors associated with (axial) pain, both in type and in strength of association, suggesting that sex-specific mechanisms may underlie pain. Absolute scores or generic cut-offs may not adequately capture clinically meaningful differences between men and women. Instead, longitudinal changes within individuals may provide more relevant insight into clinical significance. Future studies could consider multivariable models to identify sex differences in pain-associated biopsychosocial factors within axSpA while also accounting for potential confounders such as centrally acting pain medications or comorbid conditions.

This study was designed as an exploratory analysis to generate hypotheses regarding sex differences across a broad range of pain-related biopsychosocial factors in axSpA. While p-values were reported, no corrections for multiple comparisons were applied to be able to detect any associations that can be used in confirmatory research. To mitigate the risk of false positives, we focused on ES and R² differences. Recognizing the potential for spurious results, we emphasized the largest effects (ES > 0.5 and > 0.8) and interpreted our findings carefully. R² values were interpreted contextually, as even modest values may be meaningful in clinical research [53]. Most patients had longstanding axSpA, which may have influenced pain perception, coping strategies, and other biopsychosocial responses. Comorbidities were assessed with the Rheumatic Disease Comorbidity Index (RDCI), however possible other causes of chronic joint pain such osteoarthritis, unrelated to axSpA was not specifically assessed. Also, the smaller number of women in our study may have limited the detection of sex-related differences. As men more often present with r-axSpA and women with non-radiographic axSpA, observed sex differences partly reflect differences in presenting symptoms and features. Additionally, differences in PROs may not always reflect clinically meaningful differences, but could also reflect inherent sex-related differences in how PROs are interpreted or scored [9–11]. Although we focussed on sex differences, a clear distinction from gender is difficult in this kind of research, where cultural and socioeconomic factors may also play a role. Additionally, CS cannot be directly measured in humans [6], and while the CSI is one of the few validated and feasible tools available, it does not diagnose nociplastic pain. However, the CSI does aid in recognizing potential presence of CS and pain-related psychosocial factors [8].

## Conclusions

Our exploratory cross-sectional study in a standard-of-care axSpA cohort indicates a different pattern in men and women in pain-related biopsychosocial assessments. Women reported higher levels of pain related to disease activity, both in axial and peripheral regions, than men. Regarding sensory processing, women had increased pain sensitivity and enhanced pain facilitation than men. Additionally, pain-related psychosocial factors and associations with axial pain related to disease activity varied between sexes. Our results provide a basis for replication and hypotheses for further research and highlight the importance of addressing pain differently in men and women with axSpA in clinical practice.

## Data Availability

The datasets used and/or analysed during the current study are available from the corresponding author on reasonable request.

## Abbreviations

ASDAS: Axial Spondyloarthritis Disease Activity Score
ASQoL: Ankylosing Spondylitis Quality of Life
AxSpA: Axial Spondyloarthritis
BASDAI: Bath Ankylosing Spondylitis Disease Activity Index
BASFI: Bath Ankylosing Spondylitis Functional Index
bDMARDs: Biological Disease-Modifying Anti-Rheumatic Drugs
CORS: Coping with Rheumatic Stressors Questionnaire
CPM: Conditioned Pain Modulation
CRP: C-reactive Protein (indirectly implied, may be relevant)
CSI: Central Sensitization Inventory
CS: Central Sensitisation
ES: Effect Sizes
GLAS cohort: Groningen Leeuwarden Axial Spondyloarthritis cohort
HADS: Hospital Anxiety and Depression Scale
ICD-11: International Classification of Diseases, 11th Revision
IPQ-R: Revised Illness Perception Questionnaire
OA: Osteoarthritis
PCS: Pain Catastrophizing Scale
PPT: Pain Pressure Thresholds
PROs: Patient-Reported Outcomes
Q2: Question 2
Q3: Question 3
QST: Quantitative Sensory Testing
RA: Rheumatoid Arthritis
R-axSpA: Radiographic Axial Spondyloarthritis
RMDs: Rheumatic and Musculoskeletal Diseases
SEM: Structural Equation Modelling
STROBE: Strengthening the Reporting of Observational Studies in Epidemiology
TS: Temporal Summation
UMCG: University Medical Center Groningen
VAS: Visual Analogue Scale
